# Comprehensive evaluation of the safety and immunogenicity of a gene-deleted variant pseudorabies virus attenuated vaccine

**DOI:** 10.1186/s13567-022-01091-4

**Published:** 2022-09-22

**Authors:** Ling Li, Yongfeng Du, Yanbin Zhang, Pengyu Li, Xinyue Liu, Xin Zhang, Jing Li, Tong Zhang, Xin Li, Dong Xiao, Peng Liu, Peng Qi, Jin Xiao

**Affiliations:** 1 Key Laboratory of Veterinary Bioproduction and Chemical Medicine of the Ministry of Agriculture, Engineering and Technology Research Center for Beijing Veterinary Peptide Vaccine Design and Preparation, Zhongmu Institute of China Animal Husbandry Industry, Co., Ltd., Beijing, China; 2 Cahic Chengdu Machinery Factory, Chengdu, 610100 China; 3 Cahic Jiangxi Biological Pharmaceutical Factory, Nanchang, 330200 China; 4 Animal Husbandry and Veterinary Station of Wendeng District, Shandong, 264400 Weihai China

**Keywords:** Pseudorabies virus, gene deletion, attenuation, safety, immunogenicity

## Abstract

Pseudorabies virus (PRV) variant infections have caused a substantial economic impact on swine production in the absence of new powerful candidate vaccines. In this study, we developed and evaluated a gene-deleted variant pseudorabies virus (PRV)-attenuated vaccine, PRV GX-ΔTK/IES, in which the genes TK, gI, gE, US9 and US2 were deleted. During a study of innocuousness, all mice inoculated with PRV GX-ΔTK/IES survived, neither clinical signs nor pathological changes were observed, and viral genomes could not be detected in the blood and tissues. All piglets inoculated with high titres of PRV GX-ΔTK/IES remained clinically healthy, and neither fever nor clinical signs were observed. Viral detection results were negative in nasal swab samples, blood and tissue samples. Moreover, none of the cohabitated piglets seroconverted during a trial on horizontal transmission. The immunogenicity was assessed through a vaccination and challenge experiment in piglets. Piglets vaccinated with PRV GX-ΔTK/IES and the commercial vaccine were completely protected from subsequent PRV infection, and the level of immunity and protection induced by PRV GX-ΔTK/IES was better than that provided by the live commercial vaccine. Thus, PRV GX-ΔTK/IES is completely safe for both nontarget and target animals and can be regarded as a novel live gene-deleted PRV vaccine candidate.

## Introduction

First described in 1813 [1], pseudorabies (PR) is one of the most important diseases that affects animal health, and the epidemic of widespread pseudorabies virus (PRV) infection in large-scale pig farms is a potential threat in China; the virus continues to produce economic losses [2]. The causative agent, PRV, is a double-stranded, DNA-based swine virus with a genome approximately 150 kb in size and belonging to the *Varicellovirus* genus *Alphaherpesvirinae* subfamily within the *Herpesviridae* family; additionally, PRV has only one serotype [3]. PRVs have the broadest host range, including pigs, mice, sheep, rabbits, dogs, cats, and cattle [4, 5]. As the natural reservoir of the virus, pigs are also considered to be the main source of infection [6]. PRV usually infects pigs at various production phases, which results in high mortality in newborn piglets and miscarriage in pregnant sows [6]. Typical clinical signs of PRV infection, such as high fever, depression, anorexia, cough, shivering, diarrhoea, and systemic neurological symptoms, have been observed [6, 7]. Similar to other alpha-herpesviruses, PRV can establish a life-long, latent infection in the peripheral neurons of the natural host, which has been recognized as a source of infection under certain conditions [8].

Although PR has been successfully eradicated from domestic pigs in many countries, including Germany, Austria, Sweden, Switzerland, the Czech Republic, the Netherlands, Denmark, the United Kingdom, Canada, New Zealand and the United States, there have been reports of the isolation of PRV from wild boar herds in some areas that have declared eradication [9, 10]. The Bartha-K61 vaccine was introduced in China from Hungary in the late 1970s and widely used from the 1990s to 2011, and PR was well controlled on most pig farms in China. However, since late 2011, PR outbreaks have suddenly occurred in some Bartha-K61-vaccinated swine herds on many farms in China. Newly emerged PRV variants are considerably more pathogenic to susceptible pigs, mice and sheep than classic PRV strains [7, 11–13]. Previous studies have shown that the Bartha-K61 vaccine does not provide completely effective protection against PRV variant infection in sheep and piglets [11, 14, 15], although high doses of commercial Bartha-K61 protect growing pigs against lethal challenge with the PRV variant [16, 17].

PRV variant infections have had a substantial economic impact on swine production in the absence of new powerful candidate vaccines [2]. Biosecurity measures and vaccination are still the main methods for the prevention, control and elimination of PR infection in swine. Owing to the limited efficiency of current commercial vaccines in providing protection from PRV variant infections in swine, a variety of genetically engineered vaccines based on PRV variants have been developed, including inactivated gE-deleted or gE/gI-deleted vaccines [18–20], live gE/gI/TK-deleted vaccines [21–23] or gI/gE/TK/UL13-deleted vaccines [24] and subunit vaccines [25].

In the present study, we first generated a TK/gI/gE/US9/US2-deleted PRV mutant based on the PRV-GX variant strain and systematically evaluated its safety in mice and piglets. The immune efficacy of the live gene-deleted PRV strain PRV GX-ΔTK/IES was also assessed in piglets by comparison with the commercial vaccine.

## Materials and methods

### Cells and viruses

Vero and PK-15 cells were all maintained in Dulbecco’s modified Eagle’s medium (Gibco, Carlsbad, CA, USA) supplemented with 10% foetal calf serum (Gibco, Carlsbad, CA, USA) and 1% penicillin and streptomycin (Sigma‒Aldrich, St. Louis, MO, USA) at 37 °C with 5% CO_2_. The PRV-GX variant strain was isolated and identified from a case of PR in Guangxi Province of China in 2017 (unpublished) and propagated in PK-15 cells.

### Generation of plasmids

To obtain the TK/gI/gE/US9/US2-deleted PRV mutant, recombinant transfer vectors pUC-TK, pUC-TK-EGFP, pUC-IES and pUC-IES-EGFP were generated. In detail, the vector pUC was obtained by synthetic design of suitable enzyme sites based on pUC18. Then, PRV-GX genomic DNA was extracted from the infected PK-15 cells as previously described [26]. The specific primers used in our study are shown in Table 1. Next, the fragment L-arm flanking the US7 gene was amplified from PRV-GX genomic DNA using the primer pair P1F/P1R and then inserted into the plasmid pUC with the restriction enzymes E*coR*I and H*ind*III, to generate the plasmid pUC-IES-L. The fragment R-arm flanking the US2 gene was amplified using the primer pair P2F/P2R and cloned into pUC-IES-L with the restriction enzymes H*ind*III and *Mlu*I to generate pUC-IES. The plasmid pEGFP-N1-del was generated by deleting the multiple cloning sites from pEGFP-N1 (Clontech, USA) using a KOD-Plus-Mutagenesis Kit (TOYOBO, SMK-101) with the primer pair P3F/P3R. Then, the EGFP-encoding region containing the CMV immediate early promoter, EGFP gene and SV40 early mRNA polyadenylation signal was amplified using the primer pair P4F/P4R, which was cloned into pUC-IES with the restriction enzyme *Hind*III, and the resulting recombinant vector was named pUC-IES-EGFP. To further knock out the TK gene from the gI/gE/US9/US2-deleted PRV, the recombinant transfer vectors pUC-TK and pUC-TK-EGFP were generated analogously, in which the fragment TK-L-arm flanking the genes UL25 and UL24 was amplified from the PRV-GX genomic DNA using the primer pair P5F/P5R, and the fragment R-arm flanking the UL22 gene was amplified using the primer pair P6F/P6R. The four transfer vectors pUC-TK, pUC-TK-EGFP, pUC-IES and pUC-IES-EGFP were purified using a TIANprep Mini Plasmid Kit (TIANGEN Biotech Co., Ltd, Beijing, China).

**Table 1:** **Oligonucleotide primers used in this study**

Primers	Sequence (5′–3′)
P1F	G***GAATTC***GGTGGTGGCGCTGATCTCCGACCCG
P1R	CCC***AAGCTT***AGCAGGCGCGCTTGGGGTCGAGG
P2F	CCC***AAGCTT***AGACGCACGAGCTGACGCG
P2R	CG***ACGCGT***CGTGTCATCGGGTGCCAGAGCGAGAGCG
P3F	CGGTCGCCACCATGGTGAGCAAG
P3R	GCGGATCTGACGGTTCACTAAACCAGCTC
P4F	CCC***AAGCTT***TTAGTTATTAATAGTAATCAATTACGGGGTCATTAG
P4R	CCC***AAGCTT***CTAGAATGCAGTGAAAAAAATGCTTTATTTG
P5F	G***GAATTC***CGCTCCAGCGGCCGCAGCTGCTCGTCCACCTCGGCCTC
P5R	CCC***AAGCTT***GGGCGGGCCCTCGACCGCGGGCCCG
P6F	CCC***AAGCTT***ACGGCGACCACATCCGGCAGTGCGTG
P6R	CG***ACGCGT***GCCGAGGCGCACCGCCGCGCGGTAAAAGTAGTACGG
P7F	GCGACGCGCCCAACCTGACGA
P7R	GGCCCCCGAGTTCAGGTACTGGATCC
P8F	TCTGTTCGACACGGACAC
P8R	GGGATGACATACACATGGC

Restriction sites for cloning are underlined and shown in italics and bold.

### Generation of PRV GX-ΔIES and PRV GX-ΔTK/IES

PK-15 cells were infected with PRV-GX at a multiplicity of infection (MOI) of 0.1 and harvested at 24 h post-infection. Vero cells were cotransfected with 5 µg PRV-GX genomic DNA and 2.5 µg pUC-IES-EGFP plasmid with Lipofectamine^®^ 2000 (Invitrogen, Carlsbad, CA, USA). Cell monolayers were treated with DMEM containing 2% FBS and 1% low melting point agarose (Invitrogen, Carlsbad, CA, USA) when the cytopathic effects (CPEs) were observed. Recombinant viruses were screened and purified from the plaques emitting green fluorescence under fluorescence microscopy. After multiple rounds of screening in PK-15 cells, the gI/gE/US9/US2-deleted recombinant viruses expressing EGFP were obtained and named PRV GX-ΔIES-EGFP. Next, Vero cells were cotransfected with the PRV GX-ΔIES-EGFP genomic DNA and plasmid pUC-IES, and a homogeneous viral population of PRV GX-ΔIES, which did not exhibit green fluorescence emission, was screened and purified. This resulting virus mutant was verified by PCR using the primer pair P7F/P7R and sequencing. Then, the TK-deleted recombinant virus was further obtained using methods analogous to those described above based on PRV GX-ΔIES, and the resulting virus mutant PRV GX-ΔTK/IES was verified by PCR using the primer pair P8F/P8R and sequencing.

### Indirect immunofluorescence assay (IFA)

PK-15 cells infected with PRV were fixed with 50% (v/v) methanol/acetone for 30 min at −20 °C and blocked with 3% BSA (fraction V bovine serum albumin) (Roche, Mannheim, Germany) for 30 min. An anti-gB or -gE antibody as the primary antibody was applied, and the cells were incubated for 90 min at 37 °C. Anti-mouse IgG (whole molecule)-FITC (Sigma‒Aldrich-F0257, USA) as the secondary antibody was applied, and the cells were incubated for 60 min at 37 °C. Images were captured using an Olympus CK41 microscope (Olympus Corporation, Japan).

### One-step growth and plaque assay

One-step growth curves and plaque assays for the PRV-GX virus and PRV GX-ΔTK/IES virus were conducted in PK-15 cells. To generate one-step growth curves, PK-15 cells were inoculated in a 6-well plate and incubated overnight, followed by inoculation of each virus at 0.1 MOI. The cultures were harvested at 4, 8, 12, 24, 36 and 48 h post-infection, and each culture was subjected to three rounds of freeze‒thaw cycles and clarified by centrifugation at 5000 × *g* at 4 °C for 5 min. The viral titres (log_10_ TCID_50_/mL) of the clarified supernatants were assayed using the TCID_50_ assay [27].

For plaque assays, PK-15 cells were inoculated in a 6-well plate and incubated overnight, followed by inoculation of each virus in each well at 10^5.0^ ~ 10^1.0^ TCID_50_/0.1 mL with tenfold dilutions, and the PK-15 cell monolayers were treated with DMEM containing 2% FBS and 1% low melting point agarose at 1.5 h post-infection. Each well was stained with crystal violet at 96 h after virus inoculation, and the size of the plaque was determined.

### Animal experiments

#### Trial 1: innocuousness in nontarget mice

Twenty-nine six-week-old specific-pathogen-free (SPF) female BALB/c mice (Charles River, China, Beijing) were randomly divided into two groups of 12 mice and one group of 5 mice. The two groups with 12 mice were intraperitoneally inoculated with 10^3^ TCID_50_ and 10^4^ TCID_50_ PRV GX-ΔTK/IES in a 100 μL volume. The 5 mice in the control group were injected with the same volume of PBS. All mice were monitored for 14 days after inoculation. Blood samples were collected at 4, 7 and 14 days from four randomly selected mice from each group inoculated with PRV GX-ΔTK/IES, and the selected mice were humanely euthanized. Viral detection in blood and organs was carried out by PCR in accordance with the guidelines of the Chinese National Technical Standardization Committee of Animal Health GB/T18641-2018.

#### Trial 2: innocuousness in target piglets

Experiment 1: All 4 week-old weaned piglets were purchased from a pig farm in Jiangxi Province of China and were free of antigens and antibodies directed against PRV. The safety of the gene-deleted PRV recombinant virus in piglets was evaluated by injection with a single dose, two doses and a tenfold dose of PRV GX-ΔTK/IES. In detail, twenty-one piglets were randomly allocated into four groups. The first group of 6 animals was intramuscularly inoculated with a single dose of 10^6^ TCID_50_ PRV GX-ΔTK/IES in 1 mL (a single dose), the second group of 6 animals was immunized twice at a 2 week interval by the intramuscular injection of 10^6^ TCID_50_ PRV GX-ΔTK/IES in 1 mL (two doses), the third group of 6 animals was intramuscularly vaccinated with 10^7^ TCID_50_ PRV GX-ΔTK/IES in 1 mL (a tenfold dose), and the last group of 3 piglets were used as control animals and were injected with the same volume of PBS in 1 mL. For 14 days after inoculation, the animals were monitored daily for clinical signs and rectal temperatures. Clinical signs were scored as described previously [28]: (1) elevated temperature above 40 °C and below 41 °C; (2) high fever above 41 °C combined with respiratory distress; (3) ataxia; (4) convulsions; and (5) moribund state or death. Nasal swabs were collected daily, and the nasal swab plugs were submerged in 0.5 mL PBS (Gibco, Carlsbad, CA, USA) containing 200 U/mL penicillin, 200 μg/mL streptomycin, 20 μg/mL gentamicin and 5 μg/mL amphotericin B. Blood samples were collected at 3, 5, 7, 14, 21 and 28 days after inoculation. Randomly selected animals were euthanized at 3, 7 and 14 days after inoculation (one animal/time point/group), and viral detection was performed with collected tissues, including brain, heart, liver, spleen, lung, kidney, tonsils and lymph nodes. After DNA preparation (Tiangen Biotech, Beijing, China) from nasal swabs, blood and tissue samples, viral detection was carried out by PCR. Sera were tested for specific antibodies to PRV-gB and gE (gp1) antigen using blocking ELISA tests (HerdChek*AntiPRVgB or HerdChek*Anti-PRVgp1, IDEXX Laboratories, USA), as directed by the manufacturer.

Experiment 2: Ten piglets were randomly allocated into two groups. One group of 5 animals was intramuscularly inoculated in the right side of the neck with 10^7^ TCID_50_ PRV GX-ΔTK/IES in a 1 mL volume, and the remaining 5 piglets were kept as cohabitation contact animals for twenty-eight days. Clinical signs and rectal temperatures were monitored as described above. Blood samples were collected at 7, 14, 21 and 28 days after inoculation, and sera were tested for specific antibodies to PRV-gB and gE.

#### Trial 3: efficacy against challenge experiment in piglets

Thirteen piglets were randomly allocated into three groups: one group of 5 animals was intramuscularly immunized in the right side of the neck with 10^6^ TCID_50_ PRV GX-ΔTK/IES in a 1 mL volume, the second group of 5 animals was immunized with one dose of commercial Bartha-K61 vaccine, and the third group of 3 piglets were used as control animals and were injected with the same volume of DMEM. Fourteen days after immunization, all 13 piglets were oronasally challenged with 10^6.0^ TCID_50_ of PRV GX in a 1 mL volume. For 14 days after immunization and challenge, the animals were monitored daily for clinical signs and rectal temperatures, and the clinical signs were scored daily post-challenge. Serum samples were collected at weekly intervals after immunization, and PRV gB- and gE-specific antibodies and neutralizing antibodies directed against the PRV GX strain were tested. The neutralization assay was carried out in accordance with the guidelines of the Chinese National Technical Standardization Committee of Animal Health GB/T18641-2002.

## Results

### Generation and characterization of the recombinant PRV GX-ΔTK/IES virus

**Figure 1:**
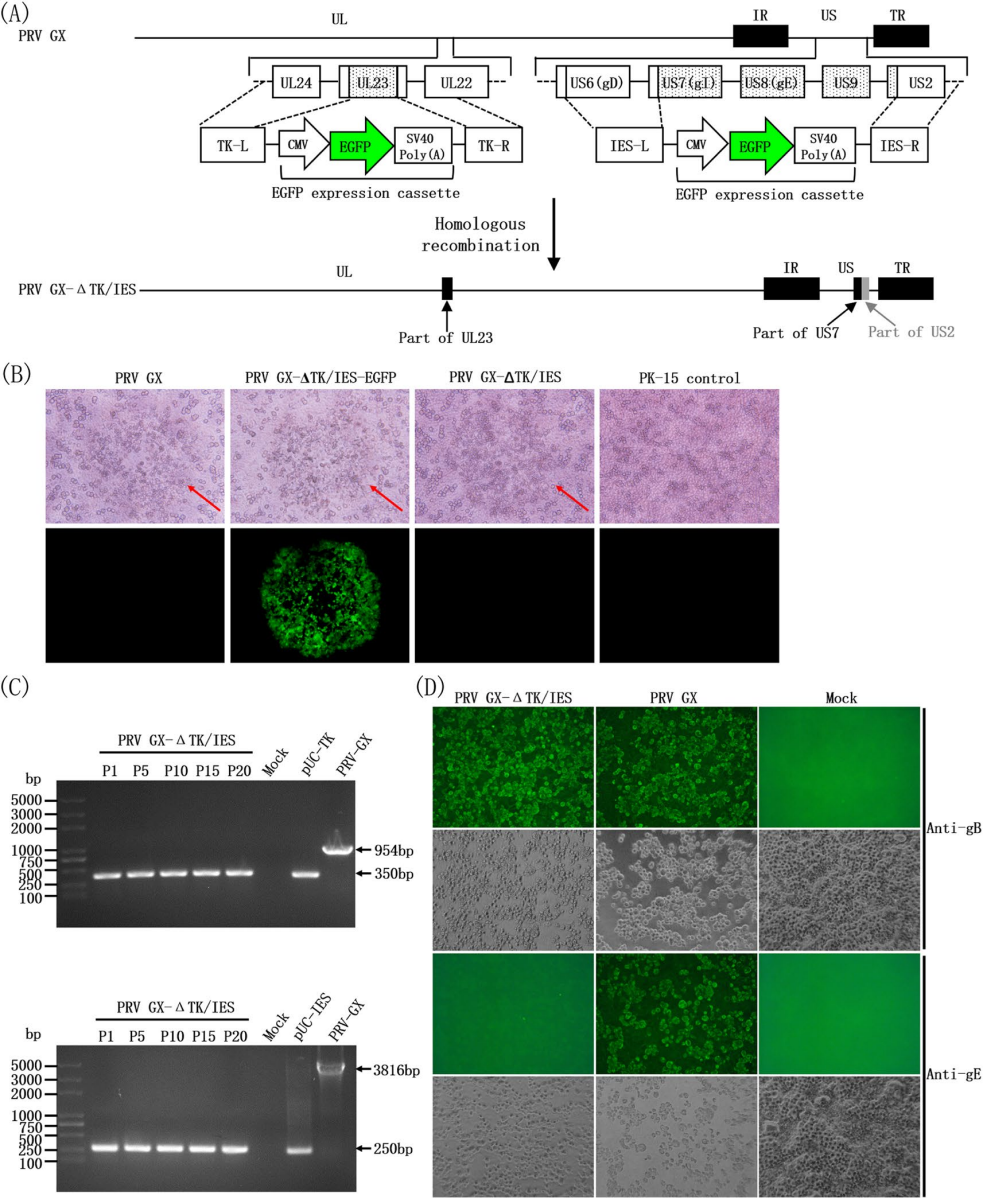
**The generation of the gene-deleted PRV GX-ΔTK/IES recombinant virus.** **A** Schematic representation of the engineered construct. The deleted regions are indicated in the full-length genome. **B** No fluorescent plaque or typically large syncytia were observed in PK-15 cells infected with PRV GX-ΔTK/IES. PK-15 cells infected with the marker virus PRV GX-ΔTK/IES-EGFP exhibited significant green fluorescence. **C** Identification and genetic stability of the recombinant PRV GX-ΔTK/IES assessed by specific PCR. A 350 bp fragment carrying the TK gene deletion and a 250 bp fragment carrying the gI/gE/US9/US2 gene deletion were amplified. Comparable DNA fragments were analysed from passage levels 1, 5, 15, and 20. PRV GX was used as the control without deletions. **D** IF analysis of PK15 cells infected with PRV GX-ΔTK/IES. The cells were stained using PRV gB- and gE-specific mAbs. The infected cell culture was anti-gB positive when treated with the PRV GX-ΔTK/IES and parental PRV GX, while the cultures were anti-gE negative when treated with the PRV GX-ΔTK/IES but anti-gE positive when treated with parental PRV GX. Control cells remained negative.

A gene-deleted PRV recombinant virus named PRV GX-ΔTK/IES was generated through homologous recombination in the presence of homology arms associated with the screening of fluorophore-tagged sequences (Figure 1A). Infection of PK-15 cells with PRV GX-ΔTK/IES-EGFP led to significantly more green fluorescence than infection with the parental viral strain PRV GX (Figure 1B), suggesting that the designed fragment containing part of the TK gene from PRV GX-ΔIES was replaced with the EGFP expression cassette. However, no fluorescent plaques with the typical large syncytia were observed in PK-15 cells infected with the resulting recombinant PRV GX-ΔTK/IES (Figure 1B). The gene-deleted mutation was further subjected to PCR using region-specific primers, followed by Sanger sequencing. As expected, correct DNA fragments in which the target gene was deleted were observed, and these constructs were compared with the parental PRV GX, a 350 bp fragment carrying the TK gene deletion and a 250 bp fragment carrying the gI/gE/US9/US2 gene deletion (Figure 1C); the sequencing results confirmed the identities. Moreover, IFA of PRV GX-ΔTK/IES and PRV GX in PK-15 cells was performed to confirm the protein expression of the gB and gE genes. The recombinant PRV GX-ΔTK/IES virus displayed the expected phenotype, which was different from the parental strain PRV GX. Proteins of the PRV GX-ΔTK/IES did not react with the PRV gE-specified mab but with the PRV gB-specified mab (Figure 1D). As a marker, the gE protein could be differentiated from the parental PRV GX and the recombinant gene-deleted PRV GX-ΔTK/IES in IFA.

**Figure 2:**
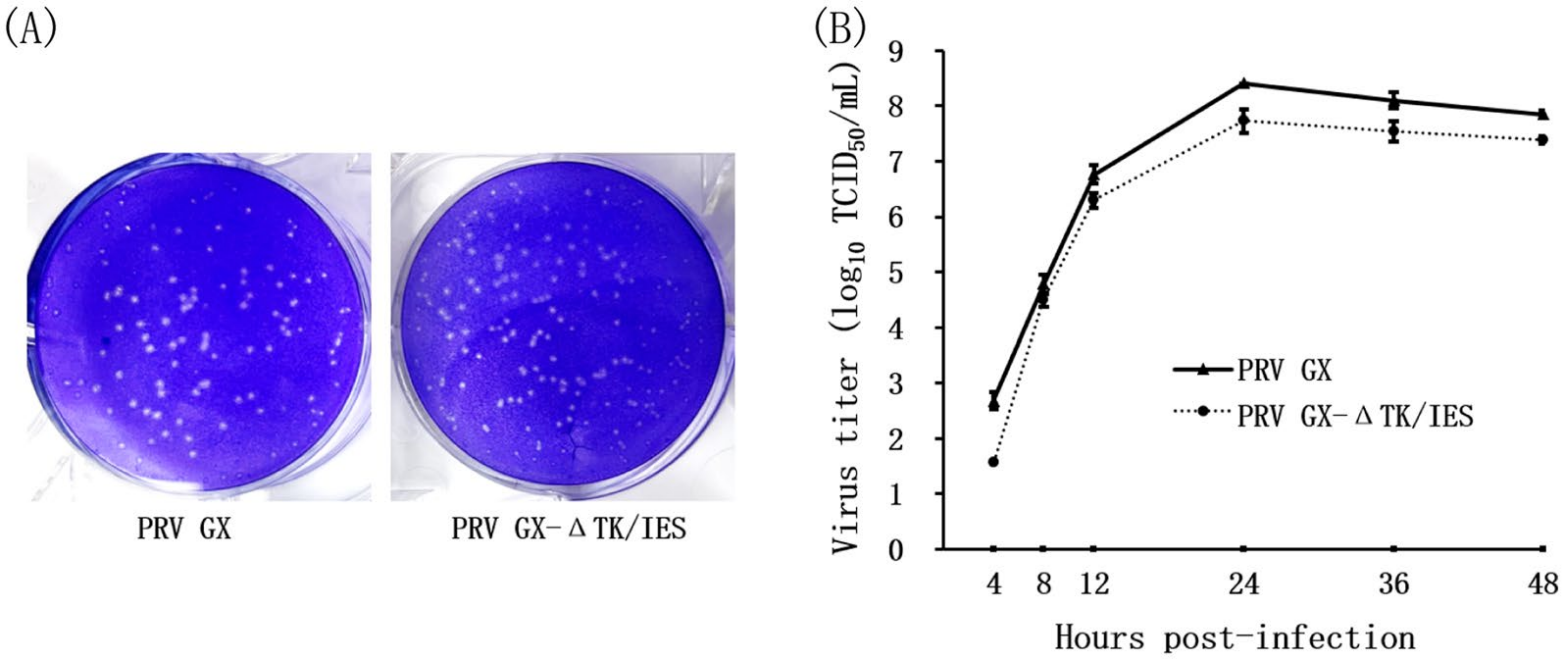
**Characterization of the gene-deleted PRV GX-ΔTK/IES recombinant virus.** **A** Crystal violet staining of PK15 cells at 96 h after infection with PRV GX-ΔTK/IES and parental PRV GX. A cytopathic effect with virus plaques was clearly visible. **B** One-step growth curves of gene-deleted PRV GX-ΔTK/IES and parental PRV GX in PK15 cells. Confluent monolayers of PK15 cells were infected at a multiplicity of infection (MOI) of 0.1, and total freeze/thaw lysates were titrated at 4, 8, 12, 24, 36, and 48 h after infection. Virus titres are described as TCID50/mL in log 10 steps.

Virus plaque formation was assessed in PK-15 cells infected with PRV GX-ΔTK/IES or PRV GX. The average size of PRV GX-ΔTK/IES plaques was slightly smaller than that of the parental strain PRV GX (Figure 2A). The kinetics of virus propagation were assessed in PK-15 cells by standardizing the inoculum at an MOI of 0.1. As shown in Figure 2B, the recombinant PRV GX-ΔTK/IES grew less than PRV GX, as shown by the reduction in virus titres. To provide relevant genetic stability data, in vitro passages (levels 1, 5, 15, and 20) of PRV GX-ΔTK/IES were obtained from PK-15 cells. The results from specific PCR amplification showed the correct DNA fragments in which the target gene was deleted (Figure 1C), and comparative sequence analysis of passage levels 1, 5, 15, and 20 revealed no nucleotide substitutions in the genome regions containing gene deletions.

#### Trial 1: innocuousness of PRV GX-ΔTK/IES in susceptible mice

**Figure 3:**
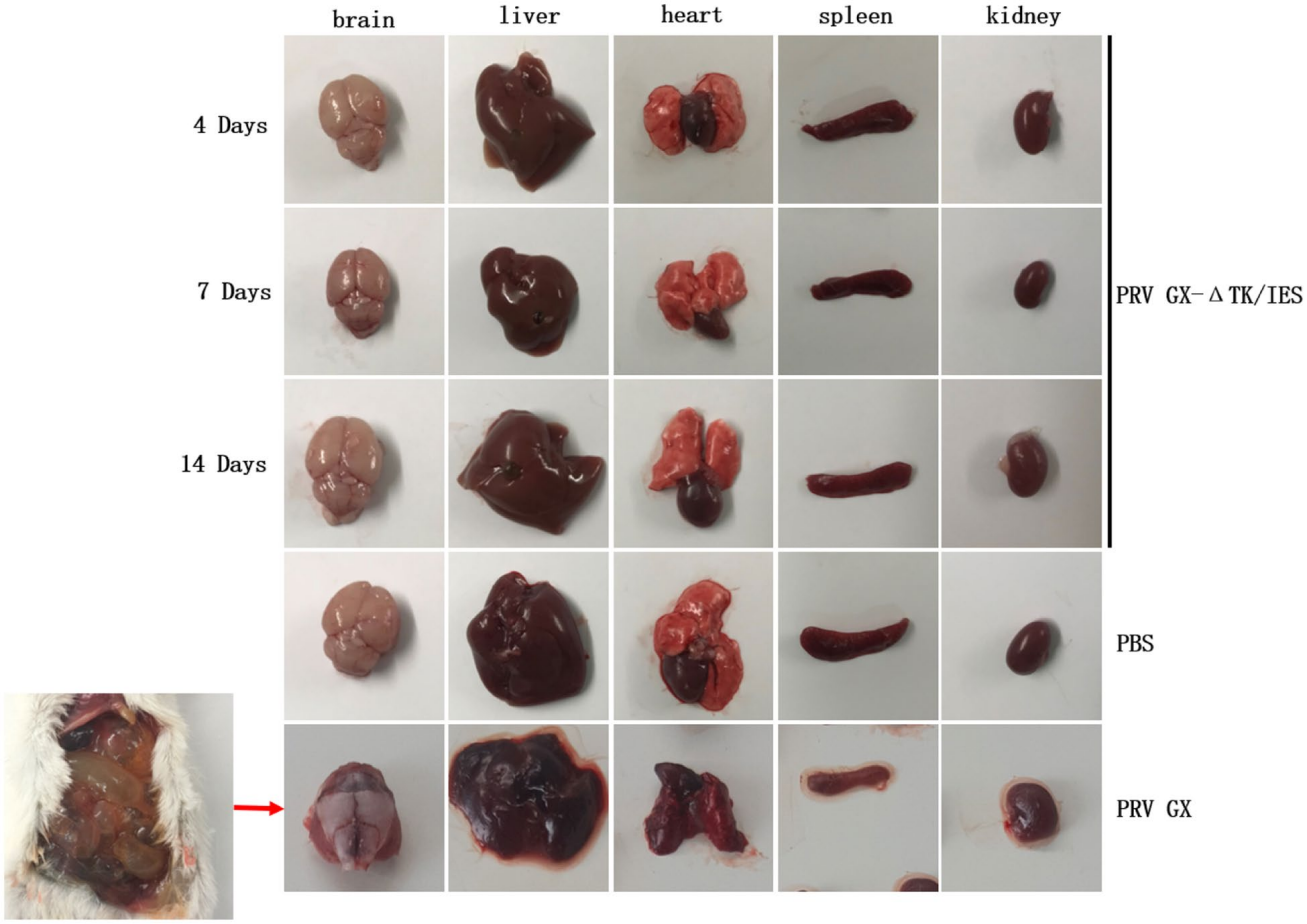
**Pathological examination of various organs of inoculated mice.** Various organ tissues from the inoculated mice, including the brain, liver, heart, spleen and kidney, were collected and used for pathological examination at 4, 7, and 14 days post-infection. The samples from the mice inoculated with virulent parental PRV GX were considered positive.

The parental PRV GX and gene-deleted PRV GX-ΔIES were highly virulent to mice, with LD_50_ 10^1.5^ TCID_50_ and 10^1.83^ TCID_50_, respectively. In contrast, all mice infected with PRV GX-ΔTK/IES were alive and healthy even at the highest dose of 10^6^ TCID_50_ (data not shown). The pathogenicity of PRV GX-ΔIES in mice was lower than that of the parental PRV GX strain. In this study, the general condition of all mice was good, and no mice died after inoculation with PRV GX-ΔTK/IES. Necropsies of mice inoculated with PRV GX-ΔTK/IES showed that the tissues and organs were normal and no obvious pathological changes in the brain, liver, heart, spleen, and kidney were observed when these animals were compared with the groups mock inoculated with PBS. Obvious pathological lesions were observed in the organs from mice infected with PRV GX (Figure 3). Viral detection performed using blood and tissue samples produced negative results in all mice inoculated with 10^3^ TCID_50_ and 10^4^ TCID_50_ PRV GX-ΔTK/IES groups at 4, 7, and 14 days post-immunization (Table 2). These data indicated the innocuousness of the gene-deleted recombinant PRV GX-ΔTK/IES virus in susceptible mice.

**Table 2:** **Viral detection in blood and tissue samples obtained from BALB/c mice inoculated with PRV GX-ΔTK/IES**

Group	Days post-inoculation	Blood	Brain	Heart	Liver	Spleen	Lung	Kidney
10^3^ TCID_50_	4	0/4	0/4	0/4	0/4	0/4	0/4	0/4
	7	0/4	0/4	0/4	0/4	0/4	0/4	0/4
	14	0/4	0/4	0/4	0/4	0/4	0/4	0/4
10^4^ TCID_50_	4	0/4	0/4	0/4	0/4	0/4	0/4	0/4
	7	0/4	0/4	0/4	0/4	0/4	0/4	0/4
	14	0/4	0/4	0/4	0/4	0/4	0/4	0/4

#### Trial 2: innocuousness of PRV GX-ΔTK/IES in piglets

**Figure 4:**
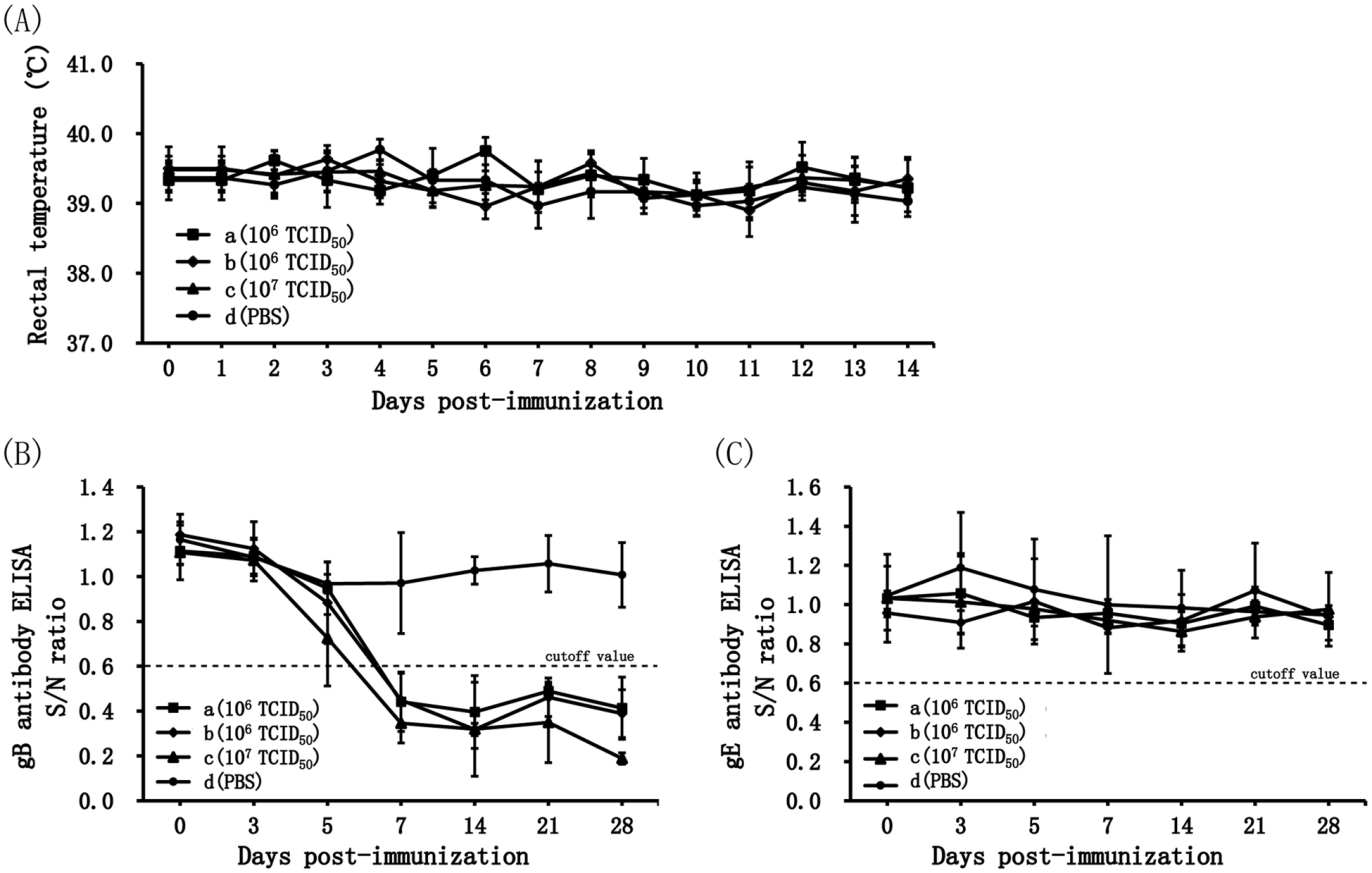
**Innocuousness of recombinant PRV GX-ΔTK/IES virus in piglets.** **A** Time course of the body temperature in the piglets after inoculation. Values represent the mean and standard deviation of the rectal temperature of the piglets in each of the groups, including those subjected to a single dose (a, 106 TCID50), two doses (b, 106 TCID50) and a tenfold dose (c, 107 TCID50), and of PRV GX-ΔTK/IES and d (PBS)-vaccinated controls. **B** PRV gB-specific ELISA antibodies after immunization with PRV GX-ΔTK/IES. **C** PRV gE-specific ELISA antibodies after immunization with PRV GX-ΔTK/IES. The levels of specific antibody to PRV-gB and gE were detected using blocking ELISA test kits. ELISA values are given as the S/N ratio. The dotted line indicates a positive antibody response (S/N ratio > 0.6), samples with S/N ratios less than 0.6 were scored as positive, and samples with ratios between 0.6 and 0.7 were scored as suspicious. Standard deviations are shown as error bars.

Following immunization with PRV GX-ΔTK/IES, all piglets remained clinically healthy and showed no side effects. Neither elevation of body temperatures throughout the observation period (Figure 4A) nor pathological lesions after necropsies (data not shown) were observed. In the nasal swab samples, as shown in Table 3, the results of the detection of viral genomes were weakly positive on Day 3 in one piglet subjected to inoculation with the single dose of 10^6^ TCID_50_, one piglet subjected to inoculation with the single dose of 10^6^ TCID_50_ twice and two piglets subjected to inoculation with the 10^7^ TCID_50_ dose. No PRV GX-ΔTK/IES viremia was detected, and the results of viral detection from tissues were negative (data not shown). In assays of PRV-specific blocking ELISA for the detection of PRV gB and gE antibodies, all piglets immunized with PRV GX-ΔTK/IES developed PRV gB-specific antibody-positive signals at Day 7 post-immunization, and a first serological reaction could be detected at Day 5 post-immunization in the 10^7^ TCID_50_-inoculated group (Figure 4B). No PRV gE-specific antibodies were detected in the sera from any immunized piglets during the entire trial period (Figure 4C).

**Table 3:** **Viral detection in nasal swab samples obtained from piglets inoculated with PRV GX-ΔTK/IES**

Group	Piglet	Days post-inoculation													
		1	2	3	4	5	6	7	8	9	10	11	12	13	14
a (10^6^ TCID_50_)	19	–^a^	–	–	–	–	–	–	–	–	–	–	–	–	–
	20	–	–	–	–	–	–	–	/^c^	/	/	/	/	/	/
	21	–	–	–	–	–	–	–	–	–	–	–	–	–	–
	24	–	–	–	–	–	–	–	–	–	–	–	–	–	–
	25	–	–	–	–	–	–	–	–	–	–	–	–	–	–
	26	–	–	+ ^b^	–	–	–	–	–	–	–	–	–	–	–
b (10^6^ TCID_50_)	27	–	–	–	–	–	–	–	/	/	/	/	/	/	/
	29	–	–	–	/	/	/	/	/	/	/	/	/	/	/
	30	–	–	–	–	–	–	–	–	–	–	–	–	–	–
	31	–	–	–	–	–	–	–	–	–	–	–	–	–	–
	32	–	–	–	–	–	–	–	–	–	–	–	–	–	–
	33	–	–	+	–	–	–	–	–	–	–	–	–	–	–
c (10^7^ TCID_50_)	11	–	–	–	–	–	–	–	–	–	–	–	–	–	–
	12	–	–	–	/	/	/	/	/	/	/	/	/	/	/
	13	–	–	–	–	–	–	–	/	/	/	/	/	/	/
	15	–	–	+	–	–	–	–	/	/	/	/	/	/	/
	16	–	–	–	–	–	–	–	–	–	–	–	–	–	–
	18	–	–	+	–	–	–	–	–	–	–	–	–	–	–
d (PBS)	14	–	–	–	–	–	–	–	–	–	–	–	–	–	–
	22	–	–	–	–	–	–	–	–	–	–	–	–	–	–
	28	–	–	–	–	–	–	–	–	–	–	–	–	–	–

Animals #29 and #12 were euthanized at 3 days post-inoculation, #20, #27, #13 and #15 were euthanized at 7 days post-inoculation.

^a^Negative sample during viral detection.

^b^Positive sample during viral detection.

^c^Animals were euthanized.

**Figure 5:**
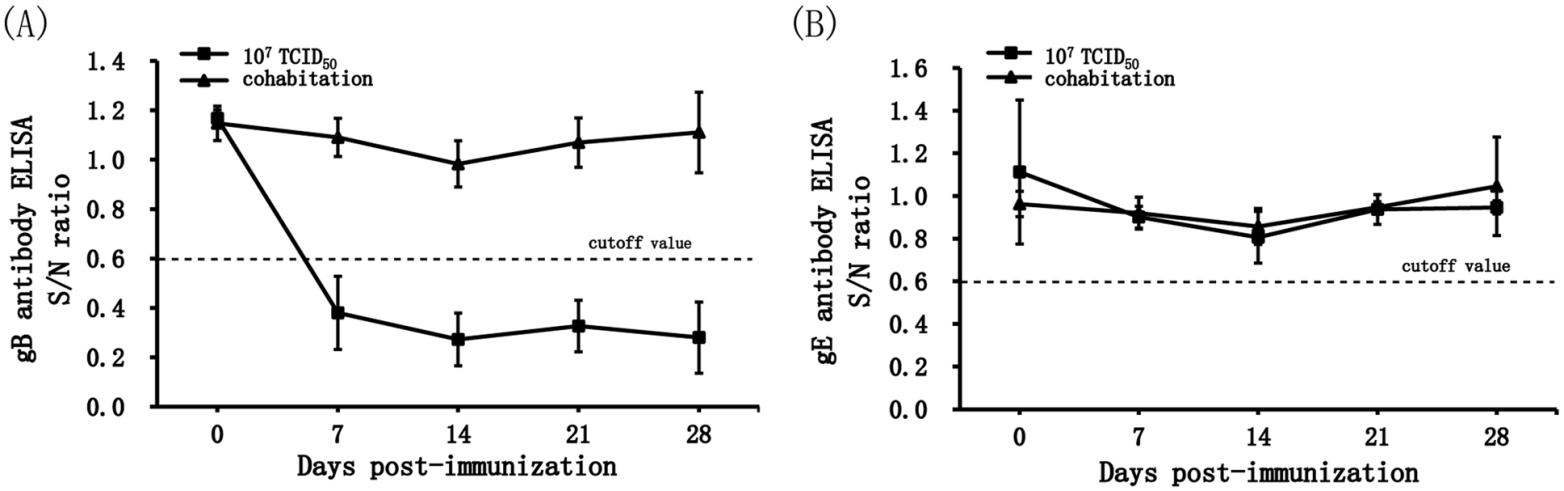
**PRV-specific ELISA antibody development after immunization with PRV GX-ΔTK/IES in the horizontal transmission trial.** **A** PRV gB-specific ELISA antibodies after immunization with PRV GX-ΔTK/IES. **B** PRV gE-specific ELISA antibodies after immunization with PRV GX-ΔTK/IES. The cohabitates were mock-immunized. The levels of specific antibody to PRV-gB and gE were detected using blocking ELISA test kits. ELISA values are given as the S/N ratio. The dotted line indicates a positive antibody response (S/N ratio > 0.6), samples with an S/N ratio less than 0.6 were scored as positive, and samples with an S/N ratio in between 0.6 and 0.7 were scored as suspicious. Standard deviations are shown as error bars.

The safety of the gene-deleted PRV GX-ΔTK/IES was further tested by horizontal transmission to sentinel piglets. None of the immunized piglets and the contact cohabitating animals displayed any clinical signs of disease or any increase in body temperature in response to PRV. Blocking ELISA analysis showed that all immunized piglets developed PRV gB-specific antibody-positive signals at Day 7 post-immunization; in contrast, the contact animals remained negative for PRV gB-specific antibodies until the end of the experiment (Figure 5A). As expected, no PRV gE-specific antibodies were detected in the sera isolated from any piglets (Figure 5B). In conclusion, none of the contact cohabitating animals exhibited seroconversion, which indicated that the transmission of PRV GX-ΔTK/IES virus from immunized piglets to other animals was limited or completely absent.

#### Trial 4: protective efficacy of PRV GX-ΔTK/IES in piglets

**Figure 6:**
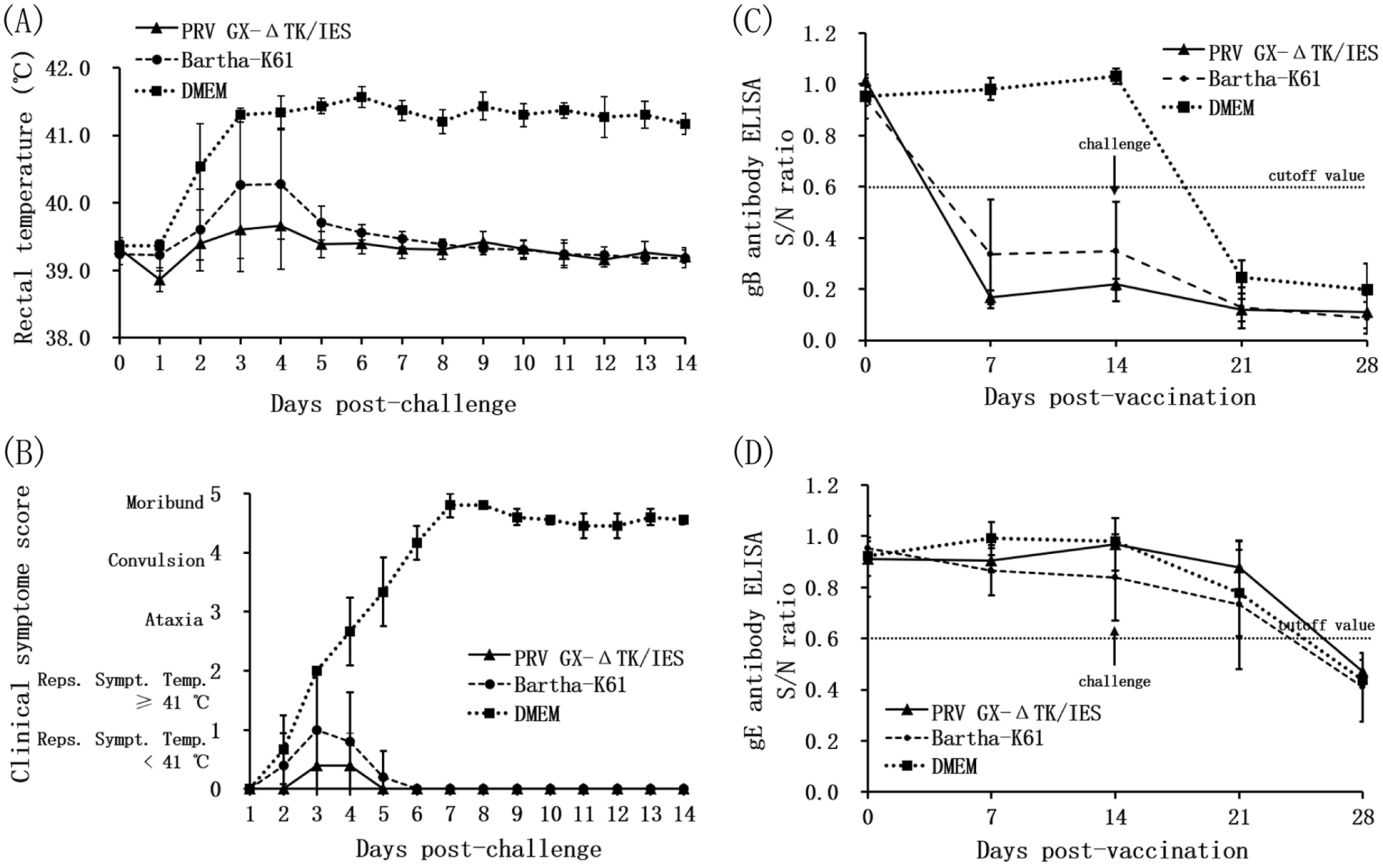
**Protective efficacy of recombinant PRV GX-ΔTK/in piglets against virulent PRV.** **A** Rectal temperature of piglets challenged with virulent PRV GX. **B** Clinical score in piglets of each group after virulent PRV GX challenge. **C** PRV gB-specific ELISA antibodies after immunization with PRV GX-ΔTK/IES and challenge with virulent PRV GX. **D** PRV gE-specific ELISA antibodies after immunization with PRV GX-ΔTK/IES and challenge with virulent PRV GX.

To evaluate the immunogenicity of the live gene-deleted vaccine PRV GX-ΔTK/IES, an immunization and challenge experiment was conducted in target piglets, and commercial Bartha-K61 was used as a vaccine control. After challenge with the highly virulent strain PRV GX at 14 days post-vaccination, the health status of the piglets previously vaccinated with PRV GX-ΔTK/IES or Bartha-K61 remained undisturbed, and the piglets were completely protected from the challenge. No fever was detected in any of the animals vaccinated with PRV GX-ΔTK/IES, while three out of five piglets in the Bartha-K61 group exhibited slight fever at 3–4 days post-challenge (Figure 6A). In contrast, all three pigs in the DMEM group displayed a significant rise in body temperature (40.9–41.7 °C) from 2 days post-challenge until death or the end of the experiment (Figure 6A) and progressively showed the typical symptoms of pseudorabies, including anorexia, depression, ataxia, convulsion and moribund state, from 3 days post-challenge, and one out of three pigs died at 7 days post-challenge. Two other pigs were moribund and had to be euthanized on Day 14 after challenge (Figure 6B).

The results obtained from blocking ELISA antibodies in serum samples after vaccination and virus challenge are shown in Figures 6C, D. All piglets vaccinated with PRV GX-ΔTK/IES or Bartha-K61 developed PRV gB-specific antibodies at 7 days post-vaccination, while piglets from the DMEM group showed seroconversion of PRV gB-specific antibodies at 7 days post-challenge (Figure 6C). PRV gE-specific antibodies were seroconverted at 7 days post-challenge in the sera isolated from all piglets of the three groups (Figure 6D). As shown in Table 4, the PRV-specific neutralization assay demonstrated that low neutralizing antibody titres were first observed at 14 days post-vaccination and progressively increased in both the PRV GX-ΔTK/IES and Bartha-K61 groups, and those in the PRV GX-ΔTK/IES group were higher than those in the Bartha-K61 group. A boost effect in the antibody response was observed after viral challenge, and a low neutralizing antibody response was detected even in the two moribund animals in the DMEM control group at 14 days after challenge.

**Table 4:** **PRV-specific neutralizing antibodies in piglets following immunization with PRV GX-ΔTK/IES and challenge with the PRV GX strain**

Group	Days post-immunization (days post-challenge)			
	7	14(0)	21(7)	28(14)
PRV GX-ΔTK/IES	< 2	5.28 ± 2.59	22.12 ± 8.14	103.90 ± 31.68
Bartha-K61	< 2	3.56 ± 3.10	18.45 ± 4.89	91.49 ± 33.87
DMEM	< 2	< 2	< 2	16.20 ± 15.54^a^

^a^Neutralizing antibody levels determined from the two animals that survived challenge with PRV GX strain.

## Discussion

Vaccination is one of the most effective ways to control PR, and among the most promising vaccine candidates are gene-deleted attenuated viruses. Apart from the protective efficacy, the development of an ideal vaccine against PR should fulfil the following three requirements: (1) safety; (2) a highly sensitive and specific serological diagnostic signature that enables differentiation of infected from vaccinated animals (DIVA); and (3) high virus titre for large-scale production of vaccine. The choice of which genes are subjected to deletion is important for the generation of attenuated recombinant viruses based on genetic engineering techniques. The PRV genome comprises two unique regions, UL and US, and two repeat sequences, IRS and TRS [3]. Some nonessential genes can be replaced without noticeable deleterious effects on virus propagation. Virulence-related genes, including UL10, UL44, UL23, UL13, UL21 and UL50, are located in the UL regions, and US7, US8, US9, US2 and US3 are located in the US regions [29–35], among which UL23 (TK), US7 (gI) and US8 (gE) are the most interesting and are usually deleted since they are critical to the virulence of the virus but have no obvious effect on its immunogenicity [12, 21]. UL23 is not essential for viral growth in cells, although the levels of UL23-negative PRV mutants were suggested to be highly attenuated in mice, rabbits and pigs [3]. As type I membrane proteins, gI and gE are required for directional spread in the nervous system [36]. When assessed with gE- and gB-ELISA, the gene-deleted recombinant PRV vaccine candidates demonstrated promising DIVA, which plays an important role in the control and eradication of PRV [2, 37–39]. Recently, some gene-deleted vaccine candidates were also artificially generated based on PRV variants using genetic engineering technologies [21–24, 40–44], in which the virulent genes TK, gI and gE were deleted. The Bartha-K61 strain is one of the best modified live vaccines obtained through extensive serial passaging of the virus on cell cultures, and molecular and genetic analyses of its complete sequence showed the deletion of a large fragment including the entire gE and US9 genes and a large portion of the gI and US2 genes [45]. Observations from previous research showed that the attenuated PRV vaccine Bartha-K61 triggered much higher type I interferon production by plasmacytoid dendritic cells than the wild-type PRV strain [46]. A high-temperature passaging PRV JS-2012-F120 strain contained a fragment in which the entire US9 gene and part of the gE and US2 genes were deleted, which was avirulent in suckling piglets in a previous study [47]. US9, a type-II transmembrane protein, interacts with neuronal proteins in the cytoplasm via its N-terminus [48]. However, as an important factor for the large-scale production of vaccines, an enhanced viral titre is needed. A previous report showed that PRV US2 gene deletion enhances viral titres but does not affect the varicosities induced by viral infection [35]. Considering the safety and to further the development of a multivalent vaccine, in our study, the PRV GX-ΔTK/IES was generated using homologous recombination methods combined with the screening of fluorophore-tagged constructs, in which the important virulent factors TK, gI and gE were knocked out while US9 and US2 were not.

PCR fragments and sequencing results showed that a 604-bp fragment in the TK gene and a 3565-bp fragment in the US region were deleted in the PRV GX-ΔTK/IES (Figure 1C), and the IFA results confirmed that the proteins of PRV GX-ΔTK/IES did not react with the PRV gE-specified mab but with that of PRV gB (Figure 1D), which could be used to differentiate naturally infected pigs from vaccinated pigs through serology (Figure 6C). Concerning replication, the generated gene-deleted PRV GX-ΔTK/IES virus displayed slightly smaller plaques and less growth than the parental PRV GX strain (Figure 2), which appeared to be consistent with the mutants expressing the US2 missense protein [35]. Analyses of the specific PCR amplification and sequencing results upon passaging in PK-15 cells confirmed the high stability of PRV GX-ΔTK/IES viruses in vitro (Figure 1C). As an important safety component, knowledge about the genetic stability of this virus in vivo still needs to be addressed in additional experiments.

Safety is a crucial parameter for a live attenuated vaccine, and genetically modified organism (GMO) safety assessments must be completed prior to a clinical trial in China; thus, safety assays in target animals and nontarget animals should be considered for live veterinary vaccines. While most reports place more emphasis on the efficacy of PR candidate vaccines, the comprehensive evaluation of the safety, especially for the attenuated PR live vaccine, is insufficient. The sensitive clinical parameter is death in mice with PRV infection. Here, we first assessed the innocuousness of PRV GX-ΔTK/IES in mice inoculated with 10^4^ TCID_50_ recombinant virus, which typically resulted in viral detection in blood and tissue samples, which was consistent with a previous study [49, 50]; these findings were accompanied by minor or absent clinical symptoms and pathological changes. PRV GX-ΔTK/IES did not lead to viraemia and negative results were observed in virological tests; no impairments in health status or shedding were observed in the investigated target piglets. Furthermore, PRV GX-ΔTK/IES was never shed or transmitted by vaccinated piglets, and all contact cohabitants remained negative in serological tests even upon high-dose inoculation (Figure 5). Interestingly, the result from the PRV gB-specific blocking ELISA test showed that a first serological reaction could be detected at Day 5 in piglets subjected to a dose of 10^7^ TCID_50,_ and a lower S/N value was observed in this group than in the 10^6^ TCID_50_ group (Figure 4B). However, the safety of PRV GX-ΔTK/IES in sows, such as vertical transmission from pregnant sows to foetuses, should be further evaluated. Since the development of multivalent veterinary vaccines is a major trend and research focus that will be further developed in the future, innocuous PRV GX-ΔTK/IES with the potential to express major immunogens from other causative agents could be a powerful vector system.

In this work, we demonstrated that the PRV GX-ΔTK/IES vaccine protected piglets from a virulent PRV variant challenge, and animals vaccinated with PRV GX-ΔTK/IES showed no clinical signs post-challenge and developed higher levels of PRV gB-specific antibodies as assessed in the blocking ELISA as well as higher levels of neutralizing antibody titres than those vaccinated with the commercial vaccine, which exhibited transient fever at 3–4 days post-challenge (Figure 6). Future work will closely examine the early onset of protection and viral shedding post-challenge, which will be particularly important in an emergency state when viral replication needs to be fully controlled and eliminated quickly. In conclusion, this study demonstrated the safety of the established vaccine in nontarget mice and target piglets and the immunogenicity and potential efficacy of the gene-deleted PRV GX-ΔTK/IES vaccine based on a variant strain.
